# Psychometric Properties of the Chinese Version of the Core Symptom Index: A Study among Chinese Parents of Children with Autistic Spectrum Disorders

**DOI:** 10.3390/ejihpe14070126

**Published:** 2024-06-26

**Authors:** Yu Chang, Bijing He, Justin DeMaranville, Nahathai Wongpakaran, Danny Wedding, Tinakon Wongpakaran

**Affiliations:** 1 Multidisciplinary and Interdisciplinary School (MIdS), Chiang Mai University, Chiang Mai 50200, Thailand; yu_chang@cmu.ac.th (Y.C.); 15268469378@163.com (B.H.); justinross.dem@cmu.ac.th (J.D.); nahathai.wongpakaran@cmu.ac.th (N.W.); danny.wedding@gmail.com (D.W.); 2 Department of Psychiatry, Faculty of Medicine, Chiang Mai University, Chiang Mai 50200, Thailand; 3 Department of Clinical and Humanistic Psychology, Saybrook University, Pasadena, CA 91103, USA; 4 Department of Psychology, University of Missouri-Saint Louis, St. Louis, MO 63121, USA

**Keywords:** psychometric property, CSI, bifactor, measurement, measurement invariance, psychological distress, ASD, autistic spectrum disorders

## Abstract

(1) Background: Parents of children with autism spectrum disorders often experience psychological distress, which can affect the quality of childcare they provide. It is crucial to screen for psychiatric symptoms among these parents. The core symptom index (CSI) is a widely recognized tool used to assess general symptoms, including depression, anxiety, and somatic issues. It has proven validity and reliability across diverse Thai populations. Given the cultural similarities between Thai and Chinese populations, the CSI has been successfully implemented within the Chinese population. Nevertheless, it is crucial to research its validity and reliability in the general Chinese population. This study aimed to investigate the psychometric properties of the Chinese version of the CSI among parents of children with autism spectrum disorders using confirmatory factor analysis (CFA). (2) Methods: A total of 794 Chinese parents raising children with autism participated in this study. All completed the CSI, along with the social inhibition subscale of the Interpersonal Problems Inventory and the Couple Satisfaction Index. Factorial validity was assessed using CFA to determine how well the bifactor three-factor model fits the data. Various structural models were compared using model fit indices. Convergent and discriminant validity were examined by exploring correlations with the social inhibition subscale and the Couple Satisfaction Index. Invariance testing of the CSI was conducted across multiple groups based on gender, age, and education using CFA. The reliability of the CSI was evaluated using McDonald’s omega coefficients. (3) Results: The bifactor model emerged as the best-fitting model for the data, suggesting that the total score of the CSI adequately represents overall psychiatric symptoms. The CSI exhibited significant correlations with the social inhibition subscale (*r* = 0.41, *p* < 0.01) and smaller correlation coefficients with the Couple Satisfaction Index (*r* = −0.16, *p* < 0.05), indicating both convergent and discriminant validity. The invariant test results support scalar invariance levels based on gender and age but only partial invariance for education. The Chinese version of the CSI demonstrated high consistency, with McDonald’s omega coefficients ranging between 0.86 and 0.95. (4) Conclusions: The bifactor model of the Chinese version of the CSI is validated, making it a suitable tool for measuring depression, anxiety, and somatization symptoms among parent(s) of children with autism spectrum disorders. Further research on other Chinese populations is encouraged.

## 1. Introduction

Autism spectrum disorder (ASD) is a significant global public health concern, imposing a substantial burden on affected families and society due to associated health challenges, especially during the COVID-19 pandemic [1,2]. Autism spectrum disorder (ASD) is a neurodevelopmental disorder characterized by social communication difficulties and repetitive, restricted behaviors and interests. As its symptoms usually appear in early childhood, raising a child with ASD not only causes distress, anxiety, and depression for caregivers but also affects family cohesion and parental relationships. Studies have shown that compared to parent(s) of children with other intellectual disabilities, parent(s) of children with autism is/are more likely to experience high levels of stress, fatigue, depression, and anxiety [3]. Additionally, since children with autism often exhibit emotional and behavioral problems, these parents face enormous caregiving burdens and pressures [4,5,6,7,8,9]. These challenges can lead to a higher likelihood of psychopathological symptoms in this/these parent(s), decreased family cohesion and parental well-being, and increased negative parenting behaviors and can hinder the effectiveness of early intervention programs [10,11]. Early detection, accurate diagnosis, and effective treatment can help alleviate their distress and improve their quality of life. Therefore, it is crucial to screen parent(s) raising children with autism for psychiatric conditions.

To identify parent(s) who are at risk of developing mental health issues, one practical approach is to utilize self-report questionnaires. The common psychiatric symptoms that can be captured by many screening scales include anxiety, depression, and somatization. One commonly used scale includes the Symptom Checklist-90 (SCL-90), which is a widely used self-report questionnaire designed to assess psychological symptoms and distress [12]. It consists of 90 items that measure 9 primary symptom dimensions: Somatization, Obsessive–Compulsive, Interpersonal Sensitivity, Depression, Anxiety, Hostility, Phobic Anxiety, Paranoid Ideation, and Psychoticism. However, due to its length and some symptoms, it has been shown to have poor discriminatory ability.

**Figure 1:**
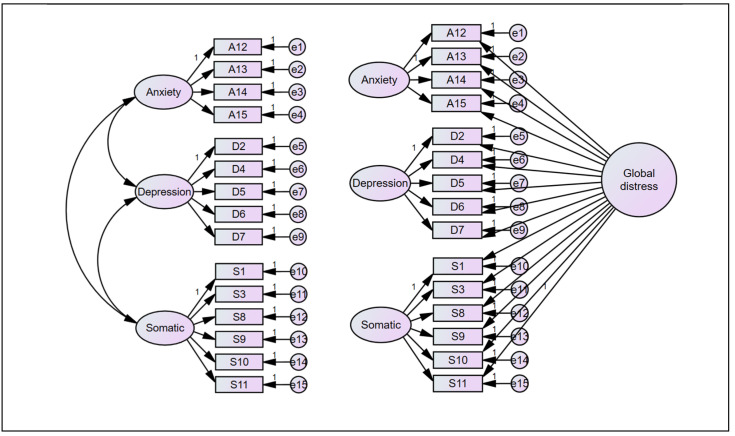
The first-order three-factor model (**left**) and the bifactor model of the Core Symptom Index (**right**). Latent variables: anxiety, depression, and somatic (somatization). Observed variables: A12–A15: anxiety indicators; D2–D7: depression indicators, S1–S11; somatic indicators. Paths and loadings: Arrows from latent and observed variables indicate factor loadings. Double-headed arrows between latent variables indicate covariances. e1–e15 represent the observed variables’ residual variances. First-order three-factor model (**left**): This model contains only specific factors without a general factor. Observed variables load onto a single specific factor, and the model includes covariances among the specific factors. Bifactor model (**right**): This model contains general and specific factors. Observed variables load onto both the general factor and their respective specific factors.

One of the brief version scales based on SCL-90 is the 18-item brief symptom index (BSI) [13,14,15]. The Chinese version of the 18-item BSI has been proven to be valid and reliable [16,17]. A study found that the three-factor and bi-factor models best fit the studied data. [16]. Another brief psychiatric symptom scale used in the Chinese population is the core symptom index (CSI), consisting of 15 measures of depression, anxiety, and somatization symptoms in epidemiologic studies. The CSI was developed initially among a Thai sample. Recent research has indicated that the CSI is psychometrically adequate. However, the first-order three-factor solution of the CSI appeared to fit the Thai sample adequately, and the bifactor model (Figure 1) was shown to fit the older Thai sample the best, allowing the CSI to be used as a single construct of psychiatric symptoms.

The CSI has been used in various populations and settings, including the general population, older residents in long-term care facilities, clinical outpatients, late adolescents, and adults [18,19,20,21,22]. The CSI has shown that the bifactor model fits best with older Thai adults. The use of the CSI among the Chinese population of parent(s) of children with ASD has some merit. First, both the CSI and BSI address similar symptoms, including anxiety, depression, and somatization, but their brevity enhances compliance. The advantage of the CSI over the BSI is that it originates from a sample with a similar Asian culture (Chinese and Thai), reducing cultural biases, particularly in somatization [23,24,25]. 

A study of the CSI among 803 older participants revealed that the three-factor model exhibited a fair level of fit, with CFI and TLI values higher than 0.9, and an RMSEA value less than 0.08. Additionally, the SRMR was less than 0.06, and the ratio of χ^2^ to *df* was greater than 3. The bifactor model of the CSI demonstrated the best-fit statistics across all models, producing a lower BIC value, indicating its statistical superiority. The common variance index showed that 61% was explained by the general factors in the bifactor model (>0.50), whereas the specific factors accounted for only 11.5% to 16.5% of the common variance. The results indicated that most items were stronger measures of general factors than specific factors.

In addition to the factor structure, the concept of measurement invariance is crucial in evaluating the quality of a measurement. In the original Thai version, measurement invariance was found to be problematic across sexes and education levels, which may be due to the older sample and the insufficient sample size. The CSI has been translated into Chinese and used with Chinese businessmen. However, the Chinese version of the CSI has yet to be evaluated in terms of its psychometric properties. 

It is important for clinicians and researchers to use the Chinese version of the CSI only if its psychometric properties have been established. These properties include factorial validity, good reliability, and established measurement invariance. Until now, there has been no study on its validity and reliability across different Chinese populations, especially among parents of children with autism. The authors hypothesize that both the three-factor and bifactor models would fit the data of parents of children with autism spectrum disorder (ASD) well. It is vital for the CSI to exhibit good reliability to ensure reproducibility and established measurement invariance to confirm that it can be equally applied to different groups, such as different genders, without biases. The authors outlined the study to test our hypotheses by exploring the factor structure of the CSI, its internal consistency, convergent and discriminant validity, and measurement invariance.

## 2. Materials and Methods

This study utilized a validation survey design involving parent(s) of children with ASD. The study involved 1030 participants. Any incomplete data, such as the absence of a medical certificate confirming ASD diagnosis in children and incomplete questionnaires, were carefully excluded. The final sample size was 794 participants aged between 23 and 45. Ethical approval for this research was obtained from the Research Ethics Committee, the Faculty of Medicine, Chiang Mai University (approval number: PSY-2566-0523). 

### 2.1. Participants 

The participants comprised general Chinese parent(s) with children diagnosed with ASD. An online survey was employed as the chosen means to generate the invitations and gather data. The inclusion criteria included (1) residing in mainland China, (2) having one or more children with a diagnosis of ASD, and (3) being able to read and write Chinese proficiently and independently complete the research questionnaire. The exclusion criteria included individuals being unable to participate online. Upon completion of the socio-demographic information, the participants proceeded with the subsequent measurements described below. The total number of participants was 794, with an equal distribution of men and women. The participants’ ages ranged from 23 to 45 years, with a mean age of 35.83 (SD: 3.26). Most participants were employed (94.1%), lived in urban areas (68.4%), had at least a high school level of education (86.9%), and had a monthly family income between RMB 3001 and 10,000 (83.7%).

### 2.2. Procedure

Parents of children with ASD were invited to participate in the study. The ASD diagnoses for the children were made by doctors in hospitals and were confirmed by medical records issued by the respective hospitals. The researchers advertised the study on major Chinese social media platforms, including WeChat, Sina, QQ, XiaoHongshu, TikTok, and Baidu Tieba, and distributed links to the online survey through these platforms. Participants who agreed to take part in the study and provided written consent received a link to the survey. Each participant independently completed the questionnaires. To protect the identities of the participants, all questionnaire data were kept confidential under ethical guidelines. The final number of participants was 794. All participants volunteered for the study and did not receive any monetary compensation.

### 2.3. Measurements 

#### 2.3.1. Core Symptom Index (CSI)

The CSI is utilized to assess general psychological symptoms. The CSI includes four items for anxiety (items 12, 13, 14, 15), five items expressing depression (items 2, 4, 5, 6, and 7), and six items measuring somatization symptoms (items 1, 3, 8, 9, 10, and 11). The respondents were instructed to provide answers based on their feelings within the previous week. The tool uses a 5-point Likert scale to rate each of its 15 items, where the responses range from 1 (rarely) to 5 (almost always). As the score increases, the level of psychopathology is interpreted as higher [20,22,26,27].

#### 2.3.2. Interpersonal Problems Inventory (IIP)—Social Inhibition Subscale

The interpersonal problems inventory (IIP-32) evaluates the challenges individuals encounter in their interactions with others [28]. The respondents assessed whether these problems arose while interacting with significant individuals in the past two weeks. The responses vary from 0 (not at all) to 4 (extremely). The inventory is divided into eight interpersonal problem subscales, such as domineering, cold, and social inhibition. The social inhibition subscale consists of four items indicating socially avoidant behavior. The Chinese version of the social inhibition subscale demonstrated a Cronbach’s alpha coefficient of 0.78 [29]. Based on its construct, social inhibition was used for the convergent validity of the CSI.

#### 2.3.3. Couple Satisfaction Index

The couple satisfaction index is a brief self-report assessment comprising 16 items designed to gauge the level of contentment within couples, irrespective of their relationship status (married, cohabiting, or dating). Scores range from 0 to 80, utilizing a 6-point Likert scale, with responses ranging from 0 (strongly disagree) to 5 (strongly agree). Higher scores denote greater levels of relationship satisfaction [30]. The internal consistency of the Chinese version of the CSI is underlined by a Cronbach’s alpha value of 0.93. Based on its construct, the couple satisfaction index was used for the discriminant validity of the CSI.

### 2.4. Statistical Analysis 

To determine the factorial validity of the core symptom index (CSI) dimensions, confirmatory factor analysis (CFA) was used to compare models to identify the best fit for the data. Different CFA models were estimated to find the optimal factor structure for the Chinese adult sample, including (a) a unidimensional model integrating all items into one factor; (b) the theoretical hypothesized three-factor model (with items 1, 3, 8, 9, and 11 loading on the somatization factor; items 2, 4, 5, 6 and 7 on the depression factor; and items 12, 13, 14, and 15 on the anxiety factor); and (c) a three-factor bi-factor model adding a global factor to the three-factor model. Model fits were assessed using chi-squares, root mean square error of approximation (RMSEA), the Tucker–Lewis index (TLI), and the comparative fit index (CFI). Conventional guidelines indicate that an RMSEA value ≤ 0.08 implies an acceptable model fit, and a value ≤ 0.05 indicates a good model fit. Meanwhile, CFI and TLI values ≥ 0.90 indicate an adequate model fit [31].

Multi-group CFAs were used to examine the measurement invariance of the CSI across gender, age, and educational level. Four types of invariances were assessed using multi-group CFA: configural, metric, scalar, and strict invariance. Configural invariance, which sets no parameters across groups, tests whether the latent variables have the same factor structure and pattern across groups, establishing a baseline model for further invariance testing. Metric invariance, based on configural invariance, sets loadings across groups to measure if each observation has the same factor loadings on the corresponding latent variables across groups. Scalar invariance sets both loadings and intercepts equivalence for each group to test if different groups have the same observation points, indicating whether there is a difference between groups. Strict invariance increases this by setting the error variance equivalence restriction. If verified, it means that differences in observed score variances among groups fully reflect differences in latent variable variances [32]. The fit indices were evaluated for each model and compared to the more restrictive model in the multi-group CFA. ΔCFI and ΔTLI less than or equal to 0.01 and an ΔRMSEA less than or equal to 0.015 indicate evidence of invariance [33]. Regarding reliability, the internal consistency was estimated using omega coefficients, with a cutoff score of >0.70 considered acceptable. Convergent validity was assessed by Pearson’s correlation coefficients on the CSI, couple satisfaction index, and social inhibition subscale to compare the magnitude of the relationship between the construct of the measuring instruments. *t*-tests were performed to examine the extent of the difference between the two constructs, signifying discriminant validity. The data were processed and statistically analyzed using IBM SPSS 26, IBM Amos version 26, and Mplus 8.11.

## 3. Results

### 3.1. Descriptive Analysis 

Table 1 shows the descriptive statistics of the CSI items, indicating that all items’ characteristics are within an acceptable range and that the scores for each item range from 0 to 4.

### 3.2. CFA Models

As shown in Table 2, except for the unidimensional model, the remaining three models demonstrated a good fit to the data (CFIs > 0.90, TLIs > 0.90). The bi-factor models provided the best fits to the data of this sample. The three-factor bi-factor model provided the best fit (χ^2^ = 440.364, *df* = 75, CFI = 0.956, TLI = 0.939, RMSEA = 0.078, and SRMR = 0.039) (Table 2).

The factor loadings were between 0.124 and 0.351 for the anxiety factor, between −0.396 and 0.214 for the depression factor, between 0.137 and 0.524 for the somatization factor, and between 0.612 and 0.805 for the general factor, confirming the bifactor three-factor solution model (Table 3). However, the factor loading of item D6 was not significant.

### 3.3. Convergent and Discriminant Validity

The results of the correlation matrix analysis of the social inhibition subscale (SI), the couple satisfaction index, and the core symptom index (CSI) and its subscales. In terms of convergent validity, there was a significant correlation between the CSI (total score) and the SI (*r* = 0.41, *p* < 0.001). On the contrary, their correlation with the couple satisfaction index was extremely low or, to some extent, almost non-existent (*r* = −0.16, *p* < 0.05). In addition, significant differences in the CSI scores between high and low levels of social inhibition were observed (*t* (658) = 8.975, *p* < 0.001), whereas significant differences in the CSI scores between high and low levels of the couple satisfaction index were not observed (*t* (705) = 1.902, *p* = 0.058). All results indicate that the CSI has convergent and discriminant validity.

### 3.4. Invariance Test 

To ensure that the three-factor bi-factor model adequately fits each group, we initially assessed its fit separately for males and females, younger and older individuals, and those with higher and lower levels of education. The results indicate a good fit of the bi-factor model for all groups. Subsequently, we tested the metric invariance model, where item factor loadings were constrained to be equal. These results suggest minimal gender differences in the model fits. Finally, scalar invariance was examined by further constraining the equal threshold across gender, age, and education groups. Scalar invariance was achieved with negligible changes in the fit indices (∆CFI ≤ 0.01, ∆TLI ≤ 0.01, and ∆RMSEA < 0.015). However, the invariance test for the education group was not fully established, revealing only partial invariance where items A15, D4, D5, S1, S8, and S9 relaxed the constraint (Table 4).

### 3.5. Reliability 

The internal consistency of the total core symptom index using the omega coefficient of the overall scale was 0.946. For the anxiety, depression, and somatization subscales, the coefficients were 0.897, 0.863, and 0.897, respectively.

## 4. Discussion

This study’s purpose was to examine the psychometric properties of the Chinese version of the core symptom index (CSI) in a sample of Chinese parents raising children with autism. To the best of our knowledge, this is the first study to investigate the psychometric properties of the CSI in the Chinese population. The bifactor three-factor solution model adequately fits the Chinese parents’ data. Convergent validity and discriminant validity were also supported as evidence of the CSI construct’s validity. Our findings, consistent with an earlier study on the Thai sample [22], confirm that the bifactor three-factor model best explains the data for the Chinese version. These findings also support the similar measurement of the BSI-18, suggesting the impact of the general factor on the uniqueness of specific anxiety, depression, and somatization symptoms [16,17]. From another perspective, the bifactor model allowed the CSI to be best conceptualized as a primarily unidimensional instrument despite the presence of some multidimensionality, a significant insight for future research. 

Regarding the measurement invariance of the CSI across groups, the findings differ from the study on the Thai sample, where the “Crying” item was not invariant for the older population, and self-blaming was not invariant across levels of education. In line with the present study, “Self-blaming” was a source of problems. The fact that the rest of the items, including “A ringing or Buzzing in the ear(s)”, “Trouble catching your breath”, “Hot or cold spells”, and “Feeling the urge to do things”, constituted non-invariance across education may be due to cultural influence. It is noted that most of the items are from somatization; even though the Thai and Chinese share similar cultures, the responses to somatization symptoms in both samples differed. Related studies have shown that somatization symptoms measured by the same CSI were significantly lower in the Thai compared to the Chinese samples (the mean ± SD was 1.04 ± 2.7 for the Thai sample and 4.38 ± 4.32 for the Chinese sample, *t* (534) = 10.38, *p* < 0.001) [18,19]. While we can use CSI despite non-achieved scalar invariance, researchers must be mindful of its limitations and use caution in its interpretation and application, particularly in cross-group comparisons. Regarding its reliability, the omega coefficient demonstrated that the CSI offers good internal consistency, consistent with the studies among Thai samples [22].

When it comes to symptoms, the most commonly reported ones include self-blame, feeling the urge to do things (anxiety), depression, feeling agitated, and crying. These symptoms indicate both anxiety and depression. It is important to note that physical symptoms are less common, suggesting that the stress experienced by the child(ren) can be effectively managed. For example, distressed parents can express their frustration. Although there are some signs of anxiety and depression, these symptoms are generally mild overall. The fact that suicidal thoughts were the least reported (less than “a little” on average) compared to other symptoms suggests that the depression experienced by the parent(s) may not have been severe.

### 4.1. Clinical Significance and Application

The CSI can evaluate parents of autistic children using the total and general scores and specific scores. The bifactor model suggests that the CSI can serve as a one-dimensional factor, enabling the application of a total score for assessing general psychological distress. Considering that somatization symptoms are how Chinese individuals may manifest psychological issues, they are influenced by cultural factors, such as ethnic identity and cultural values [34,35]. Furthermore, despite the similarities between Thai and Chinese cultures and their differences from Western culture, they are not entirely identical. Somatization items from Western scales (e.g., BSI-18) or the CSI (Thai) may not align perfectly with Chinese respondents. Therefore, it is advisable to replicate these findings in other Chinese populations to confirm whether the CSI includes somatization items that may require further modification. Based on this study’s findings, we recommend the application of the CSI in developing comprehensive mental health screening programs specifically for parents of children with autism. This tool facilitates healthcare professionals in swiftly obtaining effective results, aiding in the early detection of psychological distress in participants. Tailored support plans can then be devised, promoting timely intervention. This will enhance the well-being of caregivers, strengthen family cohesion, and contribute to more effective parenting and caregiving strategies.

### 4.2. Limitations

Be aware of the following limitations that must be addressed. Firstly, the study sample was restricted to individuals aged 23 to 45 years old and parent(s) of children with ASD. As a result, the findings may not be generalizable to other age groups or populations. Secondly, the data were collected during the COVID-19 pandemic, and the policy of home isolation in China may have influenced individuals’ responses to the items. Thirdly, modern measurement theory scholars have criticized classical test theory for the “inherent defects” of the mathematical models it is based on. Therefore, it is necessary to test the CSI according to modern measurement theory. In the future, further verification of the difficulty and discrimination of the Chinese version of the CSI in item response theory may be necessary. Lastly, the data collection did not cover the primary healthcare field, so its applicability may be limited to certain specific populations or disease conditions.

## 5. Conclusions

This study indicates that the CSI serves as a reliable and valid instrument for measuring general psychological distress among Chinese parents of autistic children, making it an effective screening tool for psychological symptoms. The bi-factor model accurately captures the underlying structure of the CSI. Moreover, the CSI demonstrates measurement invariance across diverse backgrounds, indicating that CSI scores can accurately reflect variations in psychological symptoms among Chinese parents of autistic children. Additionally, this study underscores the significance of evaluating the general factor and adopting a holistic approach to understanding parental distress rather than solely focusing on individual dimensions. Based on our findings, we recommend using the CSI in mental health screening programs for parents of children with autism. This tool helps healthcare professionals quickly detect psychological distress, allowing for timely intervention and tailored support plans that enhance parental well-being, strengthen family cohesion, and improve parenting and caregiving strategies. Further research on psychometric property should be confirmed by modern theory, such as item response theory or Rasch measurement theory. 

## Figures and Tables

**Table 1:** Descriptive statistics, skewness, and kurtosis of the CSI items (*n* = 794).

CSI Item	Mean	SD	Variance	Skewness	Kurtosis
1. Ringing or buzzing in the ear(s)	0.893	1.029	1.151	1.028	0.042
2. Suicidal idea	0.655	0.972	0.944	1.402	1.057
3. Palpitation	1.053	1.027	1.053	0.776	−0.068
4. Crying	1.340	1.130	1.275	0.455	−0.781
5. Self-blaming	1.628	1.149	1.319	0.076	−1.097
6. Feeling lonely	1.329	1.124	1.261	0.407	−0.799
7. Depressed	1.531	1.152	1.325	0.119	−1.105
8. Trouble catching your breath	0.966	1.026	1.05	0.812	−0.220
9. Hot or cold spells	0.927	1.068	1.138	0.973	0.069
10. Feeling numb or tingling	0.888	1.088	1.183	1.028	0.106
11. Fullness in the head or nose	0.967	1.064	1.130	0.808	−0.387
12. Discomfort when in a crowd	1.229	1.163	1.350	0.476	−0.944
13. Upset when being left alone	1.307	1.182	1.394	0.407	−1.017
14. Feeling agitated	1.348	1.151	1.322	0.352	−0.937
15. Feeling the urge to do things	1.543	1.187	1.407	0.106	−1.07

SD = Standard deviation.

**Table 2:** Comparison of fit indices among the CSI models.

Model	χ^2^	*df*	χ^2^/*df*	RMSEA	SRMR	TLI	CFI
Unidimensional model	1280.517	90	14.228	0.129	0.064	0.834	0.858
First-order model	667.659	85	7.854	0.093	0.050	0.914	0.930
Higher-order factor model	652.186	84	7.764	0.092	0.049	0.915	0.932
Bifactor model	440.364	75	5.871	0.078	0.039	0.939	0.956

*df* = degree of freedom, RMSEA = root mean square error of approximation, SRMR = standardized root mean square residual, TLI = Tucker–Lewis Index, CFI = comparative fit index.

**Table 3:** Standardized factor loadings for the CSI bi-factor model.

Item	Description	Anxiety	Depression	Somatization	Global
A12	Discomfort when in a crowd	0.124 **			0.805 ***
A13	Upset when being left alone	0.315 ***			0.814 ***
A14	Feeling agitated	0.300 ***			0.770 ***
A15	Feeling the urge to do things	0.351 ***			0.751 ***
D2	Suicidal idea		0.214 ***		0.705 ***
D4	Crying		−0.343 ***		0.676 ***
D5	Self-blaming		−0.396 ***		0.694 ***
D6	Feeling lonely		−0.100 **		0.809 ***
D7	Depressed		−0.506 ***		0.715 ***
S3	Palpitation			0.137 ***	0.691 ***
S8	Trouble catching breath			0.261 ***	0.750 ***
S9	Hot or cold spells			0.524 ***	0.697 ***
S10	Feeling numb or tingling			0.512 ***	0.612 ***
S1	Ringing or buzzing in the ear(s)			0.374 ***	0.649 ***
S11	Fullness in the head or nose			0.359 ***	0.690 ***

A = anxiety, D = depression, S = somatization. ** *p* < 0.01 and *** *p* < 0.001.

**Table 4:** Invariance test results of CSI’s multi-group CFA.

Model	χ^2^ (*df*)	*p*-Value	CFI	TLI	RMSEA	ΔCFI	ΔTLI	ΔRMSEA	Interpretation
Sex									
Configural	591.335 (152)	<0.001	0.948	0.928	0.060				
Metric	642.640 (177)	<0.001	0.945	0.935	0.058	0.003	0.007	0.002	Accept
Scalar	643.091 (178)	<0.001	0.945	0.935	0.057	0.000	0.000	0.001	Accept
Age									
Configural	581.098 (150)	<0.001	0.950	0.930	0.060				
Metric	637.664 (664)	<0.001	0.946	0.936	0.058	0.004	0.006	0.002	Accept
Scalar	685.727 (191)	<0.001	0.943	0.937	0.057	0.003	0.001	0.001	Accept
Education									
Configural	549.041 (150)	<0.001	0.950	0.931	0.058				
Metric	654.799 (176)	<0.001	0.940	0.929	0.059	0.010	0.002	0.001	Accept
Scalar	827.039 (191)	<0.001	0.921	0.913	0.065	0.019	0.016	0.006	Reject
Partial invariance	751.414 (186)	<0.001	0.930	0.921	0.062	0.010	0.008	0.003	Accept

Note: χ^2^ (*df*): Chi-Square value and degrees of freedom, CFI: comparative fit index, TLI: Tucker–Lewis index, RMSEA: root mean square error approximation.

## Data Availability

The datasets used and/or analyzed during the current study are available from the corresponding author upon reasonable request.
